# Microsatellite instability derived *JAK1* frameshift mutations are associated with tumor immune evasion in endometrioid endometrial cancer

**DOI:** 10.18632/oncotarget.9414

**Published:** 2016-05-17

**Authors:** Ellen Stelloo, Marco A. Versluis, Hans W. Nijman, Marco de Bruyn, Annechien Plat, Elisabeth M. Osse, Reinhardt H. van Dijk, Remi A. Nout, Carien L. Creutzberg, Geertruida H. de Bock, Vincent T. Smit, Tjalling Bosse, Harry Hollema

**Affiliations:** ^1^ Department of Pathology, Leiden University Medical Center, Leiden, The Netherlands; ^2^ Department of Gynecologic Oncology, University Medical Center Groningen, University of Groningen, The Netherlands; ^3^ Department of Radiation Oncology, Leiden University Medical Center, Leiden, The Netherlands; ^4^ Department of Epidemiology, University Medical Center Groningen, University of Groningen, Groningen, The Netherlands; ^5^ Department of Pathology, University Medical Center Groningen, University of Groningen, Groningen, The Netherlands

**Keywords:** JAK1, microsatellite instability, endometrial cancer, antigen presentation machinery, HLA class I

## Abstract

*JAK1* frameshift mutations may promote cancer cell immune evasion by impeding upregulation of the antigen presentation pathway in microsatellite unstable endometrial cancers (ECs). This study investigated the *JAK1* mutation frequency, its functional implication in immune evasion and its prognostic significance in microsatellite unstable EC. Microsatellite instability and three microsatellite repeats within *JAK1* were analyzed in 181 ECs. Sixty-two (34%) ECs showed microsatellite instability, of which 22 (35%) had a *JAK1* mutation. LMP7, TAP1 and HLA class I protein expression and the presence of CD8-positive T-cells were analyzed in the microsatellite unstable ECs. *JAK1* mutant microsatellite unstable ECs showed impaired upregulation of LMP7 (*P*=0.074) and HLA class I (*P*<0.001), validated using RNAseq data of the TCGA. TAP1 expression and presence of CD8-positive T-cells were not related to *JAK1* mutations. In 198 additional microsatellite unstable ECs, the *JAK1* mutation frequency was confirmed but no prognostic significance was found. For, *JAK1* wildtype (n=135, 72%) and mutant (n=52, 28%) ECs, 10-year recurrence free rates were 84% and 77% (*P*=0.301). These observations show that *JAK1* mutations are highly frequent in microsatellite unstable EC, not associated with survival, but are associated with impaired upregulation of LMP7 and HLA class I and may therefore facilitate immune escape.

## INTRODUCTION

About 30% of endometrial cancers, predominantly of endometrioid histology, can be molecularly characterized by microsatellite instability (MSI). MSI is a hypermutable phenotype caused by the loss of DNA mismatch repair (MMR) activity mostly due to sporadic *MLH1* promoter hypermethylation [1]. Tumors that exhibit this phenotype have numerous insertions and deletions also in coding microsatellites causing frameshift mutations and loss of protein function. The coding microsatellite-containing genes frequently affected by MSI are believed to be involved in progression of MSI tumors [2]. Some target genes, such as *BAX,* are altered in diverse MSI tumor types (e.g. colorectal- and ovarian cancer), whereas others, such as *JAK1*, have a very restricted occurrence in MSI endometrial cancers [3–6]. MSI endometrial cancers show a remarkably high number of *JAK1* frameshift mutations that may have clinical implications [4, 5].

JAK1 plays a role in the JAK/STAT pathway, which is activated by cytokines such as IFNγ, that influence several cellular processes such as cell growth and immune response [7–9]. Ren *et al.* have shown that *JAK1* mutant gynecological cancer cell lines were defective in interferon gamma (IFNγ) induced STAT1 tyrosine phosphorylation and thereby impede upregulation of antigen processing machinery components such as LMP2 and TAP1 [10]. Impaired antigen processing and presentation due to hindered expression of LMP and/or TAP proteins are associated with lack of HLA class I upregulation and resistance to cytotoxic T-cell mediated lysis [11, 12]. HLA class I expression has been reported as a prognostic marker in endometrial cancer patients [13–15] and upregulation of HLA class I was frequently impaired in MSI endometrial cancers [14, 16]. The high rate of *JAK1* mutations in MSI endometrial cancer is suggestive of an adaptation favoring tumor survival by blocking the JAK/STAT pathway activity, and impeding an adequate immune response.

MSI tumors exhibit a high number of somatic mutations that could facilitate an immune response by presentation of neo-antigen-epitopes in the context of HLA class I molecules. Programmed death 1 expressed on cytotoxic T-cells is a checkpoint involved in immune suppression. Checkpoint inhibitors, as potential mechanism for T-cell activation, recently showed promising results in treatment of mismatch repair deficient tumors independent of tumor origin [17]. However, *JAK1* mutations and other mechanisms involved in impeding antigen presentation and expression of antigen processing machinery components in MSI endometrial cancers may interfere with new treatment regiments for MSI tumors such as the programmed death 1 inhibitor pembroluzimab. [17, 18].

In this study, MSI and *JAK1* mutation status were analyzed in a study cohort of 181 tissue samples of endometrial cancer patients with the aim to evaluate that the *JAK1* locus is frequently affected by MSI, and to determine its functional implication in immune evasion by analyzing expression of antigen presenting machinery components and the presence of cytotoxic T-cells specifically in MSI endometrial cancers. Finally, the effect of *JAK1* mutation status on survival was evaluated in a large independent cohort of 198 MSI endometrial cancer patients with mature long-term follow-up from the PORTEC-1 and -2 clinical trials [19, 20].

## RESULTS

Of the 181 endometrial cancers from the study cohort, MSI was detected in 62 (34%) cases, in nine cases MSI status remained unclear due to technical failure. Twenty-two (35%) MSI endometrial cancers had a *JAK1* frameshift mutation, mainly at position K860, whereas only 3 of 110 (3%) MSS endometrial cancers had a *JAK1* mutation (*P*<0.001, Supplementary Table S1). Two of these three *JAK1* mutant MSS cases showed focal loss of MLH1 protein expression in part of the tumor as a result of *MLH1* promoter hypermethylation. There were no significant differences in age, FIGO stage, differentiation grade or tumor type between *JAK1* wildtype and mutant MSI endometrial cancers (Table 1). However, *JAK1* mutations were associated with deeper myometrial invasion (*P*=0.030; odds ratio 3.500, 95% confidence interval 1.102-11.116).

**Table 1:** Clinicopathological characteristics of 58 MSI endometrial cancers of the study cohort and 187 MSI endometrial cancers of the PORTEC cohort according to *JAK1* mutation status

	Study cohort			PORTEC cohort		
	*JAK1* wildtype n=36 (62.1%)	*JAK1* mutation n=22 (37.9%)	*P-value*	*JAK1* wildtype n=135 (72.2%)	*JAK1* mutation n=52 (27.8%)	*P-value*
**Age**			1.000			0.914
<60 years	18 (50.0)	11 (50.0)		19 (14.1)	7 (13.5)	
>60 years	18 (50.0)	11 (50.0)		116 (85.9)	45 (86.5)	
**Tumor type**			0.430			0.534
Endometrioid	35 (97.2)	22 (100)		134 (99.3)	52 (100)	
Serous	1 (2.8)	0 (0.0)		1 (0.7)	0	
FIGO (2009)^*^			0.650			0.270
I	22 (61.1)	11 (52.4)		135 (100)	52 (100)	
II	3 (8.3)	4 (19.0)		0	0	
III	10 (27.8)	5 (23.8)		0	0	
IV	1 (2.8)	1 (4.8)		0	0	
**Grade**			0.885			0.725
1	13 (36.1)	7 (31.8)		77 (57.0)	33 (63.5)	
2	14 (38.9)	10 (45.5)		31 (23.0)	10 (19.2)	
3	9 (25.0)	5 (22.7)		27 (20.0)	9 (17.3)	
Myometrial invasion*			0.030			0.172
<50%	21 (55.3)	6 (28.6)		45 (33.3)	12 (23.1)	
>50%	15 (41.7)	15 (71.4)		90 (66.7)	40 (76.9)	

* 1 missing value in the study cohort

**Figure 1:**
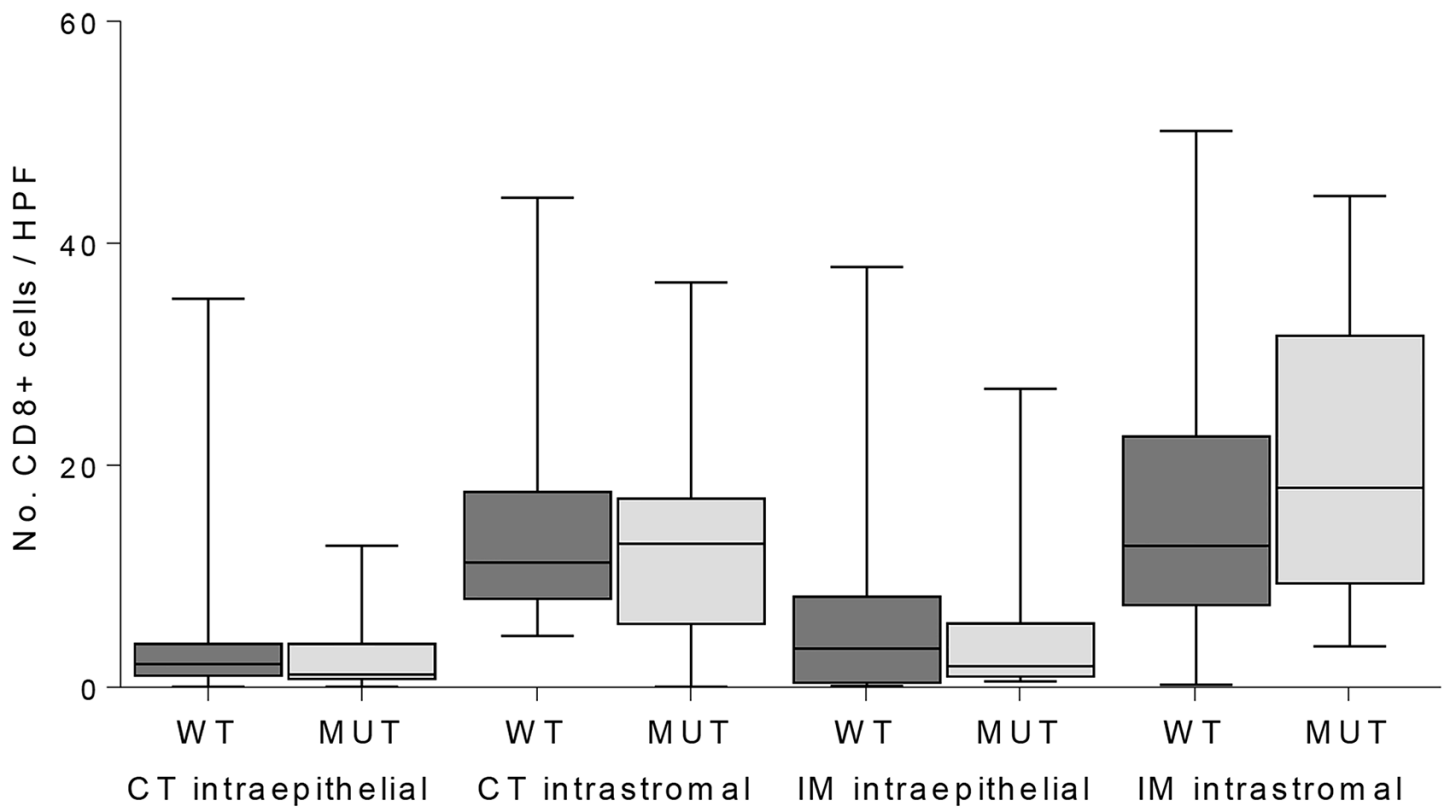
Quantification of CD8-positive T-cells in intraepithelial and intrastromal components in the center of the tumor (CT) and invasive margin (IM) in JAK1 wildtype (WT) and mutant (MUT) MSI endometrial cancers

The functional implication of JAK1 in tumor immune evasion was analyzed by expression analysis of TAP1, LMP7, HLA class I and presence of CD8-positive T-cells in the MSI endometrial cancers (Table 2). Distribution of TAP1 expression was similar for both *JAK1* wildtype and mutant (*P*=0.151). Upregulation of LMP7 was impaired in *JAK1* mutant tumors, although not statistically significant (*P*=0.074). Upregulation of HLA class I was significantly impaired in *JAK1* mutant tumors (*P*<0.001). The expression of HLA class I was related to LMP7 expression in contrast to TAP1 (*P*=0.001 vs. *P*=0.381). Presence of CD8-positive T-cells was not related to *JAK1* mutation (Figure 1).

**Table 2:** Expression of antigen processing machinery components TAP1, LMP7 and HLA class I in *JAK1* wildtype and mutant MSI endometrial cancers

	*JAK1* wildtype n=36 (62.1%)	*JAK1* mutation n=22 (37.9%)	*P*-value
TAP1^*^			0.151
Impaired/Normal	24 (68.6)	18 (85.7)	
Upregulated	11 (31.4)	3 (14.3)	
LMP7^*^			0.074
Impaired/Normal	8 (22.9)	10 (45.5)	
Upregulated	27 (77.1)	12 (54.5)	
**HLA class I**			<0.001
Impaired	7 (19.4)	12 (54.5)	
Normal	3 (8.3)	6 (27.3)	
Upregulated	26 (72.2)	4 (18.2)	

* 1 missing value

**Figure 2:**
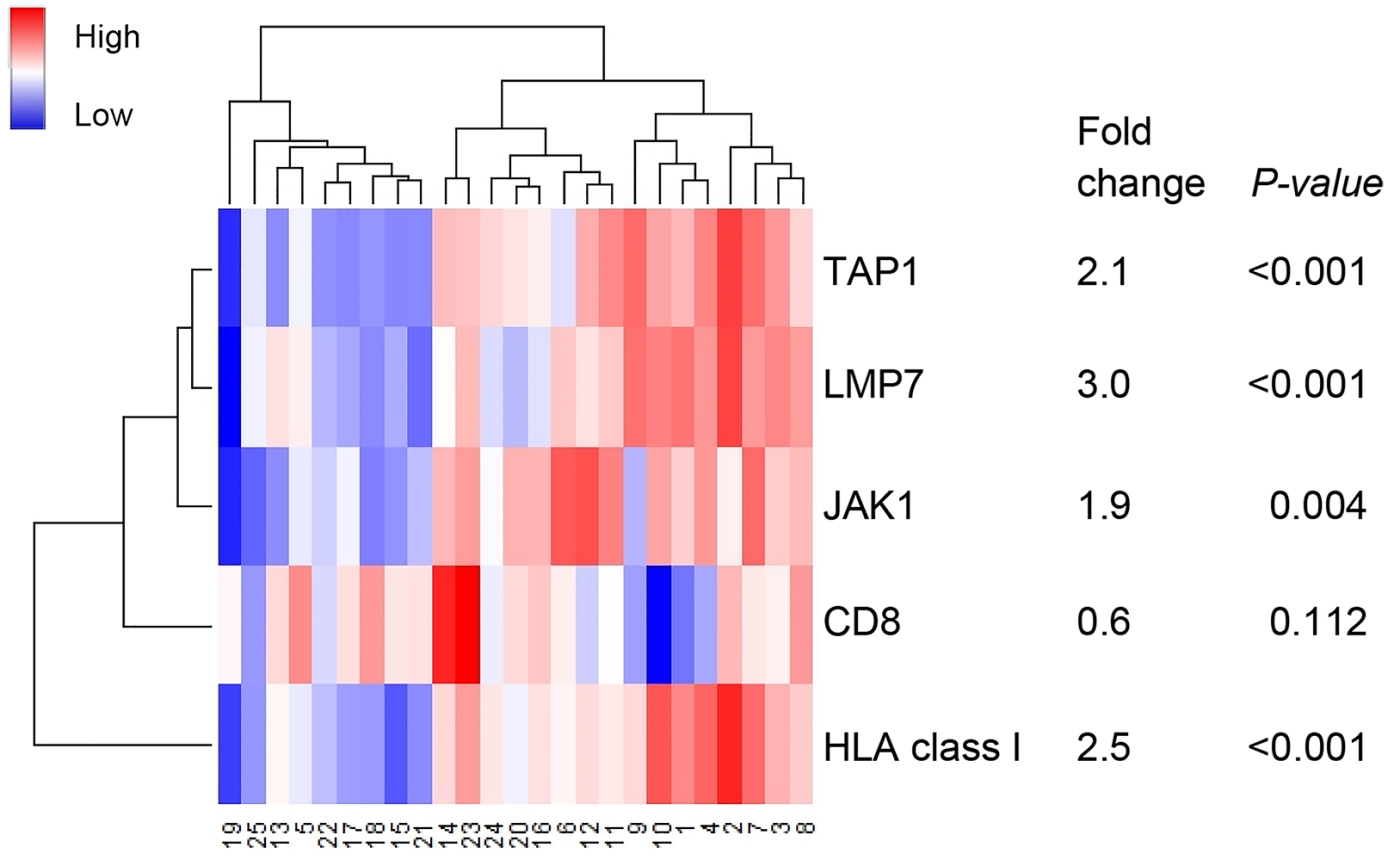
Heatmap of RNA expression of genes encoding for antigen machinery components Case numbers 1-12 represent JAK1 wildtype and 13-25 JAK1 mutant MSI endometrial cancers. A broader analysis independent of MSI status was previously shown by Kim *et al*.

In order to validate these findings in an independent cohort, RNAseq data was used from 25 TCGA MSI endometrial cancers with analyzed DNA slippage events. The 13 *JAK1* mutant endometrial cancers showed significantly lower expression of TAP1 (2.1-fold, *P*<0.001), LMP7 (3.0-fold, *P*<0.001), and HLA class I (2.5-fold, *P*<0.001) in comparison to *JAK1* wildtype endometrial cancers (Figure 2). Consistent with the results in our study cohort, *JAK1* mutation status did not correlate with CD8 expression (*P*=0.112).

**Figure 3:**
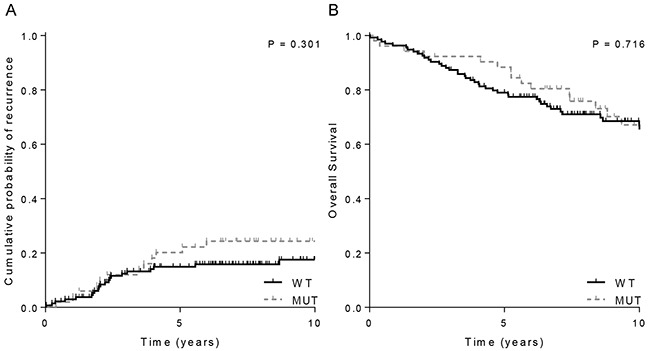
Clinical outcome of 198 MSI endometrial cancer stratified by JAK1 mutations status **A.** Overall recurrence rate and **B.** Overall survival. WT= wildtype; MUT=mutant

Two patient cohorts derived from the PORTEC-1 and -2 randomized trials with MSI early-stage endometrial cancers (n=198) with long-term mature follow-up data were used to investigate a possible prognostic effect of *JAK1* frameshift mutations. In this independent cohort, fifty-two (28%) of MSI endometrial cancers had a *JAK1* mutation (Supplementary Table S1). No significant differences were found between *JAK1* mutation status and clinicopathological characteristics, and the association of *JAK1* mutation and deep myometrial invasion could not be confirmed (Table 1). This discrepancy could be explained by the fact that this cohort consisted of significant more tumors with deep myometrial invasion compared to the study cohort (Supplementary Table S2). For *JAK1* wildtype and mutant endometrial cancers, 10-year recurrence free rates were 84% versus 77%, respectively (*P*=0.301) and 10-year overall survival was 64.4% and 63.5% (*P*=0.716) (Figure 3). Neither did subanalysis (e.g. in grade 3 cancers, or when analyzing only pelvic and distant recurrences) show a significant difference in outcome between *JAK1* wildtype and mutant endometrial cancer patients. Survival analysis within the TCGA microsatellite unstable endometrial cancers with known *JAK1* mutation status (n=30) showed neither a survival benefit for *JAK1* wildtype tumors (data not shown).

## DISCUSSION

This research shows that frameshift mutations in *JAK1* frequently and almost exclusively occur in MSI endometrial cancers. *JAK1* mutations were associated with impaired upregulation of antigen presenting machinery proteins LMP7 and HLA class I. The association of *JAK1* mutations with limited expression of the antigen presentation pathway was validated using RNAseq data of the TCGA MSI endometrial cancers. Impaired upregulation of HLA class I expression interferes with tumor lysis by cytotoxic T-cells, and therefore *JAK1* mutations may facilitate an immune escape. However, no effect was observed of *JAK1* mutation status on recurrence rate and overall survival in a large independent cohort of 198 MSI endometrial cancers. These findings suggest a functional role for *JAK1,* although with no prognostic value which suggests that *JAK1* mutations are pivotal to cancer initiation and/or maintenance, in an intriguing tissue-specific manner.

The overall *JAK1* mutation frequency of 28% in our large series of MSI endometrial cancers is in line with findings from two recent smaller studies [4, 5]. Both studies also showed that *JAK1* frameshift mutations in MSI tumors are tissue specific and significantly less important in colorectal cancers as compared with endometrial cancers. Similarly, Ren *et al.* identified *JAK1* frameshift mutations mainly in gynecological cancers, primarily in endometrial and cervical cancer, when sequencing more than 3,000 tumors from various human tissues [10]. Altogether, the occurrence of *JAK1* mutation specifically in endometrial cancer is suggestive of a positive selection for this mutation in endometrial cancer.

To date, there is very little evidence for the functionality of *JAK1* frameshift mutations in endometrial cancer. An *in vitro* study with one endometrial cancer and two ovarian cancer cell lines harboring *JAK1* frameshift mutations demonstrated that *JAK1* mutations impede STAT1 posphorylation and upregulation of antigen presenting machinery components LMP2 and TAP1 [10]. Kim *et al.* have shown that *JAK1* mutations were associated with hampered JAK/STAT signaling and lymphocyte activation [4]. These findings suggest that *JAK1* mutations have a negative effect on tumor immune surveillance. Our study now confirmed impaired upregulation of LMP7 and HLA class I with no effect on TAP1 expression and the number of CD8-positive T-cells in *JAK1* mutant endometrial cancer tissue samples. The findings on protein expression were validated using RNAseq data of TCGA MSI endometrial cancers, except for TAP1. TAP1 gene expression may not be equal to its protein expression because of the small number of cases with RNAseq data (n=25) or due to the (post-)translational process into proteins. The lack of correlation between *JAK1* mutations and CD8-positive T-cells might be explained by the fact that recruitment and migration of T-cells do not rely on recognition of peptides presented by HLA class I molecules. In addition, previous studies have also shown a high correlation between LMP7 expression and HLA class I expression, but not with LMP2 or TAP1/2 [21, 22]. This further strengthens the argument that *JAK1* mutations favor immune escape via the JAK/STAT signaling, although, this study did not evaluate the activation status of the JAK/STAT signaling by phospho-STAT1 expression.

The association of *JAK1* mutations and lack of HLA class I upregulation in MSI endometrial cancers with no effect on clinical course was an unanticipated finding of our study. Bijen *et al.* and Yakabe *et al.* reported impaired upregulation of HLA class I as a prognostic marker for survival in endometrial cancer patients [13, 15]. Of note, a large proportion of HLA class I negative endometrial cancers are MSI [14], therefore, separate analysis of MSI and MSS tumors would be of interest to determine the prognostic impact of HLA class I. This study indirectly showed no effect of HLA class I expression on survival via the *JAK1* mutation status in a large cohort of MSI, early-stage endometrial cancers. HLA class I expression was not analyzed on the precious PORTEC tissue samples as this will only validate previous findings of Ren et al., the study and TCGA cohort and the fact that *JAK1* mutations have no effect on survival will remain. However, the relatively good prognosis of this cohort of early-stage cancers needs to be taken into account. The previous studies were performed in heterogeneous groups of endometrial cancers consisting of different histologic subtypes and different FIGO stages [13–15]. Therefore, the prognostic role remains to be determined in higher risk endometrial cancers. These results, in contrast to the finding that *JAK1* mutations favor immune evasion, may suggest a lack of negative selection of *JAK1* mutations specifically in endometrial cancer.

Considering the process of immune surveillance in cancer [11, 12], it is likely that *JAK1* mutant tumors are still recognizable to cells of the innate immune system (natural killer cells) or that *JAK1* wildtype tumors have encountered other mechanisms to evade immune-mediated killing. However, low numbers of natural killer cells that lack an association with HLA class I expression were observed in endometrial cancer (unpublished data by Versluis *et al*.). On the contrary, IFNγ production might be responsible for a CD4-positive T-cell-mediated antitumor immunity [23].]. Furthermore, *JAK1* wildtype tumors may not demonstrate a survival benefit because of other strategies to dampen immune response such as upregulation of anti-apoptotic molecules, expression of immune-inhibitory ligands or secretion of immunosuppressive cytokines. Nevertheless, our findings imply that *JAK1* mutations may exert in part its oncogenic effects by immune escape, but we cannot exclude other contributions of *JAK1* in the JAK/STAT signaling [7].

Better understanding of the antigen-specific immune responses and tumor microenvironment may guide immunotherapy. Recently, immune checkpoint blockade were reported as promising therapies for tumors with a high mutational load, including mismatch repair deficient endometrial cancers, as a result of an increased neo-antigen specific T-cell response [17]. *JAK1* mutations in MSI endometrial cancers may interfere with the T-cell response due to impaired HLA class I or PD-L1 expression [17, 18]. In melanoma, one patient without a clinical response to PD-1 blockade and increased T-cell response showed a loss of function *JAK1* mutation that unables PD-L1 upregulation. All other fifteen melanoma patients did not show genetic alterations in the interferon receptor signaling pathway [18]. These limited data suggest that *JAK1* mutations may be used as negative selective predictive biomarker for immune blockade therapy. However, additional studies are required on the immune microenvironment of *JAK1* mutant endometrial cancers (e.g. PD-L1 expression).

We have identified a high frequency of *JAK1* mutations in MSI endometrial cancers in two relatively large series of MSI endometrial cancers. In addition, *JAK1* mutations may have a negative effect on tumor immune surveillance due to lack of HLA class I upregulation on the cell surface. It must be noted that it remains unclear why *JAK1* mutations are limited to gynecological cancers and mainly to MSI endometrial cancer. However, *B2M* frameshift mutations, also leading to immune escape via loss of HLA class 1 expression, frequently occur in MSI colorectal cancer and are a rare phenomenon in MSI endometrial cancer [14, 24]. In addition, Xiong *et al*. showed that JAK1 inhibition is associated with cell cycle arrest and apoptosis in colorectal cancer [25]. No effect on cell viability upon IFNγ was found in *JAK1* mutant gynecological cell lines [10]. In conclusion, we confirmed the remarkably high frequency of *JAK1* mutations and associations with impaired upregulation of antigen presenting machinery components in MSI endometrial cancers, which suggest a functional role for *JAK1* in an intriguing tissue-specific manner.

## MATERIALS AND METHODS

### Patients

DNA analysis and immunohistochemical staining was performed on a study cohort of 181 endometrial cancers with endometrioid histology, treated at the University Medical Center Groningen between 1985-2004 or at the University Medical Center Leiden between 2000-2013. Classification and grading was done according to the World Health Organization criteria and staging was according to FIGO guidelines (2009). No follow-up data was available for this study cohort.

To validate our findings regarding the *JAK1* mutation frequency in MSI endometrial cancer, an independent cohort of 198 MSI early-stage endometrial cancers derived from the randomized PORTEC-1 and -2 clinical trials was used [19, 20]. To estimate the impact of *JAK1* mutation on survival the same cohort of 198 MSI endometrial cancers was used [26].

### MSI and *JAK1* mutation status

DNA was isolated as previously described [27]. The MSI status of each tumor was determined using the Promega MSI analysis system (version 1.2, Promega). Tumors with instability in at least two markers were defined as being high-frequency MSI whereas those showing no instability or instability in one marker were classified as being stable (MSS). *JAK1* frameshift mutations (k142fs, p430fs, k860fs) were detected by Sanger sequencing. The following primers were used: exon 5-F: 5′-GTCACATCTGGGTCCCCTTTGCCAC-3′, exon 5-R: 5′-CACAAACTCCAGCTTCTCCTGGGC-3′, exon 9-F: 5′-GTCGAGGAGGCCTTG TCCTTTGTGTC-3′, exon 9-R: 5′-ACACGGGCTCTCTGCACACC-3′, exon 19-F: 5′-GTATCGACTGCCTTTCACTCTG-3′, exon 19-R: 5′-CTTACCTCTCCCAAGTCACGG-3′.

### Immunohistochemistry

Formalin-fixed paraffin embedded 4-μm tissue sections of MSI endometrial cancers (n=58) with sufficient tumor tissue were immunohistochemically stained for expression of LMP7, TAP1, HLA class I (HCA2 and HC10) and CD8 (marker of cytotoxic T-cells). Sections were deparaffinized in xylene, rehydrated in graded concentration of ethanol and microwave antigen retrieval was performed in 10 mM citrate pH 6.0 (LMP7, TAP1), 10 mM Tris/1 mM EDTA pH 9.0 (LMP7, TAP1, HCA2, HC10 and CD8) before staining. Endogenous peroxidase was blocked by incubation in a 0.3% hydrogen peroxide solution. LMP7 and TAP1 were stained using anti-LMP7 mouse monoclonal 1B3 (Novus Biologicals) and anti-TAP1 rabbit polyclonal H300 (Santa Cruz) as primary antibodies by incubation overnight at 4°C (dilution 1:100 and 1:50 respectively). HLA class I was stained using HCA2 and HC10 as previously described [16]. Antigen-antibody reactions were visualized using 3.3′-diaminobenzidine (DAB) and slides were counterstained with hematoxylin.

### Evaluation of immunohistochemistry

Two observers blinded to clinicopathological features, MSI and *JAK1* mutation status independently evaluated the stained slides. Expression of LMP7 and TAP1 was scored using a semiquantative scale as described previously [13, 28]. This score is based on the percentage of cells stained and the intensity of staining. The percentage of cells was scored on a 6 point scale with 0 for 0%, 1 for 1-5%, 2 for 5-25%, 3 for 25-50%, 4 for 50-75% and 5 for 75-100%. The intensity was scored on a 4 point scale with 0 indicating absence of staining and 3 indicating strong staining. The expression of LMP7, and TAP1 was categorized in impaired (score 0-2), normal (score 3-6) and upregulated (score 7-8) expression. For analysis of HLA class I expression, the percentage of tumor cells with membranous HCA2 and HC10 staining was quantified as previously described [16]. The expression of HLA class I was defined as follows: impaired HLA class I expression: less than 5% of tumor cells expressing both HCA2 and HC10, normal HLA class I expression; less than 5% of tumor cells expressing either of the markers, and upregulated HLA class I expression: 5% or more expressing both markers.

The number of CD8-positive T-cells was calculated using the average number of stained cells in 8 fields at 40x magnification. The average was calculated for four locations: intraepithelial at the tumor center, intraepithelial at the invasive margin, intrastromal in the tumor center and intrastromal at the invasive margin. For statistical analysis values for CD8 were dichotomized using the median as a cut off.

### The cancer genome atlas (TCGA) RNAseq data

Details of the TCGA RNAseq analysis have been previously reported [1]. Level 3 RSEM normalized RNA data profiled using the Illumina HiSeq RNAseq v2 were retrieved at the TCGA data portal. MSI events, differences in length of microsatellites, in 30 MSI EC patients were reported by Kim et al [4]. In total, 25 MSI endometrial cancers with both RSEM normalized and DNA slippage event data were informative for analysis.

### Statistical analysis

*JAK1* mutation status was compared between cases with and without microsatellite instability using Chi-square tests. Similarly, Chi-square tests were used to detect differences in expression of LMP7, TAP1, HLA class I and number of CD8-positive T-cells below or above the median for cases with and without *JAK1* mutation. The non-parametric Mann-Whitney test was used for all comparisons of continuous data and Spearman's rho to analyze correlation between variables. RNAseq data was visualized by unsupervised clustering using RStudio.

To evaluate the impact of *JAK1* mutation status on survival in MSI endometrial cancer patients that participated in the randomized PORTEC-1 and -2 clinical trials, time-to-event analyses were calculated from the date of randomization to date of recurrence (vaginal, pelvic and/or distant recurrence) or to date of death (overall survival); patients who were alive and without recurrence were censored at the date of last follow-up. Survival curves were calculated using the Kaplan—Meier method with log-rank test.

Analyses were performed using SPSS (v20, IBM statistics, Chicago, IL, USA).

## SUPPLEMENTARY TABLES


