# Supplementary material for: Microsatellite instability derived JAK1 frameshift mutations are associated with tumor immune evasion in endometrioid endometrial cancer
Source: Oncotarget. 2016 May 17;7(26):39885–93. doi: 10.18632/oncotarget.9414 (PMC5129978; doi:10.18632/oncotarget.9414)
Supplement: Supplementary file 1 [file oncotarget-07-39885-s001.pdf]

Microsatellite instability derived *JAK1* frameshift mutations are associated with tumor immune evasion in endometrioid endometrial cancer

SUPPLEMENTARY TABLES

Supplementary Table S1: *JAK1* mutation frequency in 181 endometrial cancers and 198 MSI endometrial cancers

n=181*	MSI n =62 (36.0%)	MSS n =110 (64.0%)	*P-value*
***JAK1* mutation status**			<0.001
Wildtype	40 (64.5)	107 (97.3)	
K142fs	1 (1.6)	1 (0.9)	
P430fs	8 (12.9)	0	
K860fs	11 (17.8)	2 (1.8)	
P430fs & k860fs	2 (3.2)	0	
* Failed analysis for 9 cases			
n=198*	**MSI n=187 (%)**		
***JAK1* mutation status**			
Wildtype	135 (72.2)		
K142fs	1 (0.5)		
P430fs	7 (3.7)		
K860fs	40 (21.4)		
K142fs & K860fs	1 (0.5)		
P430fs & k860fs	3 (1.6)		

* Failed analysis for 11 cases

Supplementary Table S2: Comparison of the clinicopathological characteristics of the MSI endometrial cancers included in the study cohort and PORTEC cohort

	Study cohort n=62 (%)	PORTEC cohort n=198 (%)	*P-value*
**Age**			
<60 years	30 (48.4)	27 (13.6)	0.000
>60 years	32 (51.6)	171 (86.4)	
**Tumor type**			
Endometrioid	61 (98.4)	197 (99.5)	0.384
Serous	1 (1.6)	1 (0.5)	
**FIGO (2009)***			
I	33 (57.9)	198 (100)	
II	7 (12.3)	0	0.000
III	15 (26.3)	0	
IV	2 (3.5)	0	
**Grade**			
1	21 (33.9)	116 (58.6)	0.560
2	27 (43.5)	44 (22.2)	
3	14 (22.6)	38 (19.2)	
**Myometrial invasion***			
<50%	29 (47.5)	63 (31.8)	0.025
>50%	32 (52.5)	135 (68.2)	

* missing values in the study cohort
